# Nodules ulcerating into comedones overlying an indurated plaque

**DOI:** 10.1016/j.jdcr.2023.10.016

**Published:** 2023-12-01

**Authors:** B. Matthew Kiszla, Anna S. Riess, Ayodele O. Adelanwa, Peter G. Pavlidakey, Lauren C.S. Kole

**Affiliations:** a Marnix E. Heersink School of Medicine, University of Alabama at Birmingham, Birmingham, Alabama; b Department of Dermatology, University of Alabama at Birmingham, Birmingham, Alabama

**Keywords:** cyst, milia, milia en plaque, radiotherapy

## Case presentation

**Fig 1:**
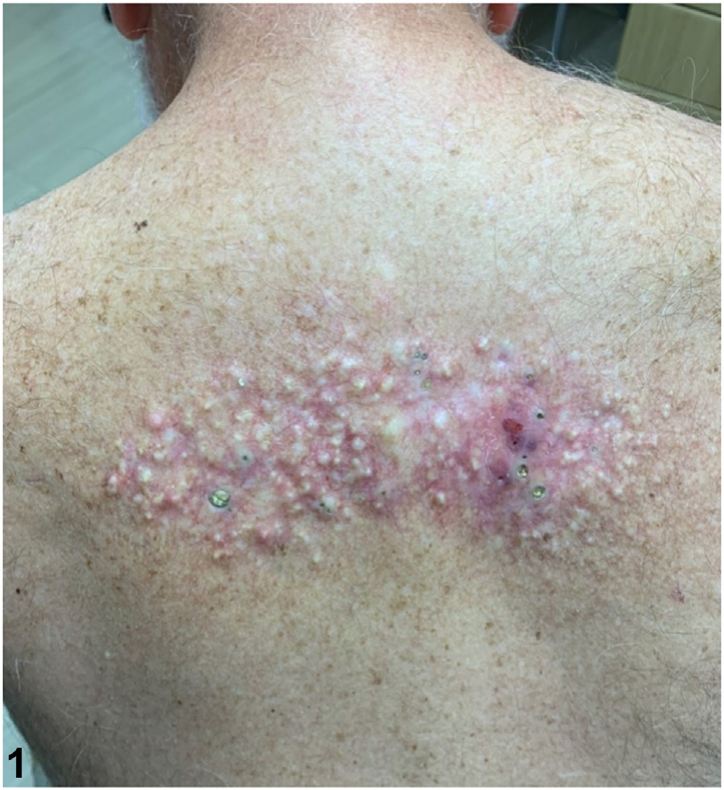

**Fig 2:**
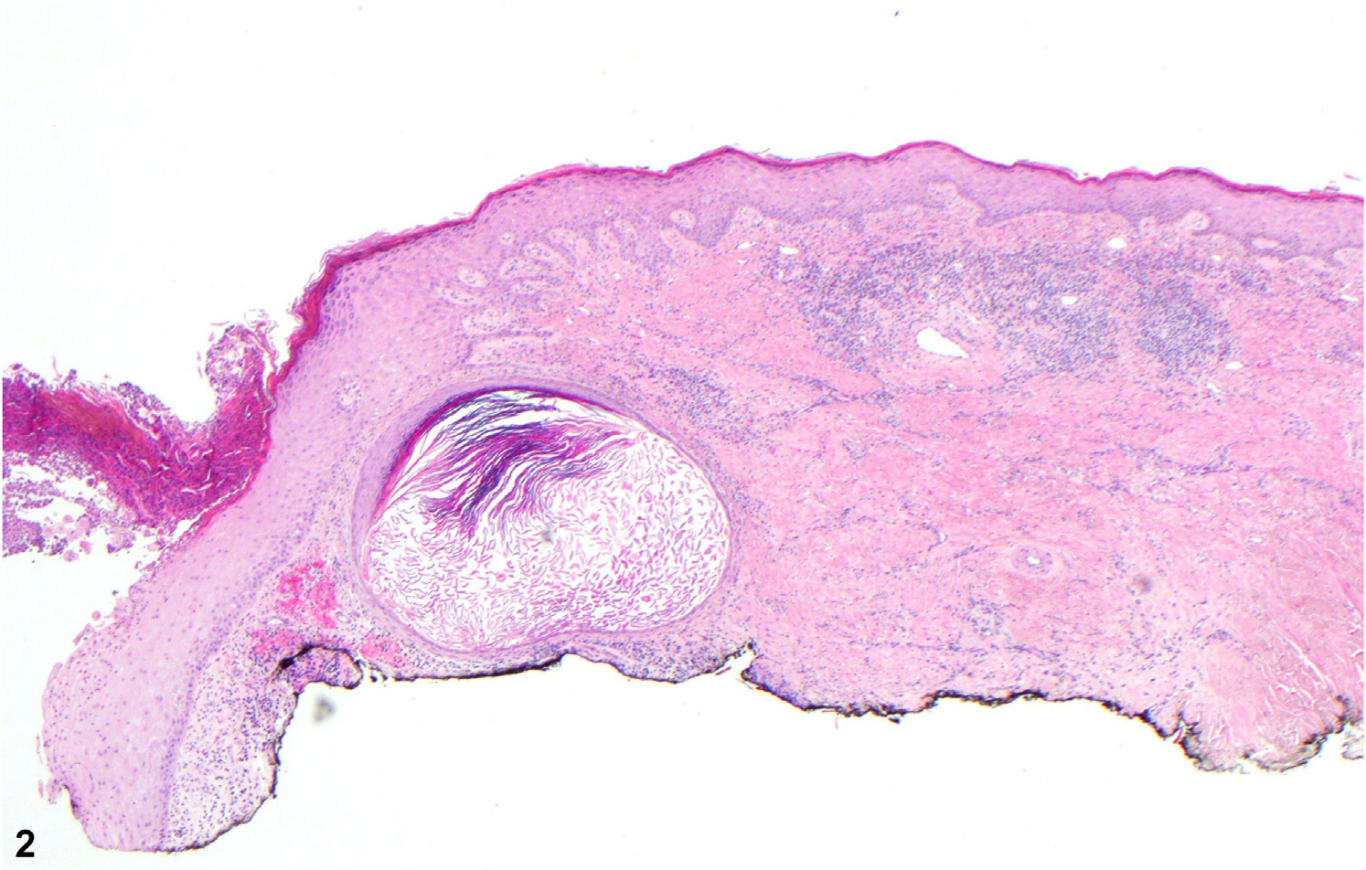

A 71-year-old male presented with a multidecade history of an indurated, diffusely telangiectatic plaque on his superior back studded with white nodules; their ulceration yielded open comedones (Fig 1). The patient had a preceding history of radiotherapy for non-Hodgkin lymphoma 30 years prior at the same site but not of trauma nor topical drug application. The patient had a history of atopic dermatitis, effectively treated with dupilumab, and nonmelanoma skin cancer. Hematoxylin and eosin stain of a shave biopsy was significant for keratinaceous cysts with stratified squamous linings surrounded by lymphoplasmacytic inflammatory infiltrates (Figs 2 and 3). Grocott methenamine silver and acid-fast staining revealed no microorganisms.

**Figure:**
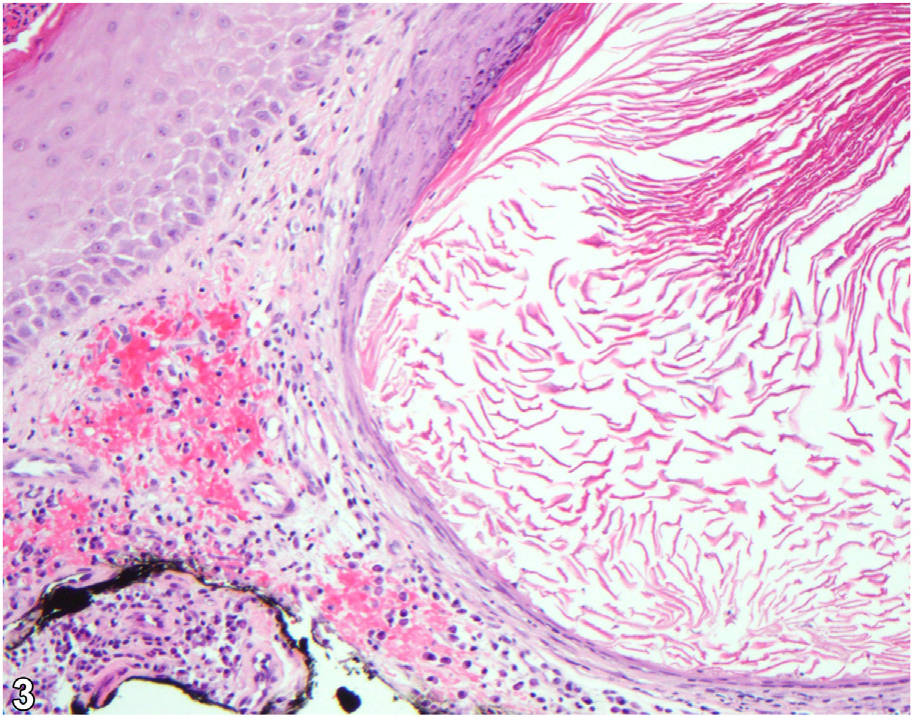

**Question 1: What is the most likely diagnosis?**

A. Basal cell carcinoma
B. Chloracne
C. Favre-Racouchot syndrome
D. Milia en plaque
E. Nevus comedonicus

**Answers:**

**A.** Basal cell carcinoma – Incorrect. Although the patient had a history of nonmelanoma skin cancer, and basal carcinomas often present as slow-growing erythematous papules with telangiectatic activity on sun-exposed skin, the presence of keratinaceous nodules and the modest degree of ulceration relative to the lesion’s large size and long length of activity favored other etiologies clinically. Histologically, one should expect a palisading basal epithelium with mitotic figures superficial to a mucinous stroma.
**B.** Chloracne – Incorrect. Though chloracne may present with nodules and open comedones, typically on the malar cheeks, retroauricular head and neck, axillae, and scrotum, its development is known to be driven by past exposures to halogenated aromatic compounds, not radiotherapy. Additionally, the indurated and elevated, plaque-like background of the lesion would be atypical of this etiology, which may have a scarred and atrophic background.
**C.** Favre-Racouchot syndrome – Incorrect. Favre-Racouchot syndrome presents with nodules and open comedones, typically on the periorbitum, and though it is often the result of chronic exposure to sunlight, it is known to develop after radiotherapy as well. However, a background of diffuse elastosis or actinic damage would be expected, not an indurated, erythematous plaque.
**D.** Milia en plaque – Correct. Milia en plaque describes the rare finding of densely grouped subepidermal keratinous cysts over an erythematous plaque. Some case reports have suggested a role for electromagnetic radiation in the pathogenesis of this condition^1^, ^2^, ^3^; given that milia are thought to result from the obstruction of hair follicles or eccrine sweat ducts, radiotherapy may not only induce genetic damage to follicular stem cells but may promote occlusive structural changes, akin to radiation-induced strictures, to these adnexa as well.
**E.** Nevus comedonicus – Incorrect. Though nevus comediconus presents as coalescing groups of open comedones on the head, neck, axillae, and/or scrotum, these lesions classically present before adolescence with a dyspigmented, not an erythematous and telangiectatic, background.

**Question 2: At what anatomical site does this lesion classically present?**

A. Conchal bowl
B. Eyelids
C. Malar face
D. Periauriculum
E. Superior chest

**Answers:**

**A.** Conchal bowl – Incorrect. Involvement of the conchal bowl of the ear by a rash has been considered nearly pathognomonic for discoid lupus erythematosus, but other entities with that localization can include primary cutaneous amyloidosis, atopic dermatitis, and seborrheic keratoses. Milia en plaque has been documented as affecting the conchal bowl, but this is not quite its classic localization.
**B.** Eyelids – Incorrect. Classic milia often involve the eyelids as part of a larger distribution also including the nasal tip in neonates and the periorbitum and infraorbital ridge in adolescents and adults. On the other hand, milia en plaque has historically been more closely associated with a different anatomical site.
**C.** Malar face – Incorrect. Skin changes affecting the malar face classically result from photosensitive rashes including systemic lupus erythematosus and rosacea. While classic milia often involves the malar face, especially the skin overlying the zygomatic bone, milia en plaque has been thought to preferentially involve a different anatomical site.
**D.** Periauriculum – Correct. Milia en plaque has historically been documented at anatomical sites adjacent to the ear, leading it to be initially thought of as a primarily periauricular process.^4^ Continued documentation, however, has suggested localization to sites of prior insults, including those on the trunk and extremities.^1^^,^^3^^,^^4^
**E.** Superior chest – Incorrect. Skin changes involving the superior chest, typically in a “V-shaped” distribution, classically result from photosensitive rashes including poikiloderma of Civatte and dermatomyositis.

**Question 3: What wavelength of electromagnetic radiation would be most effective in the treatment of this condition?**

A. 308 nm
B. 532 nm
C. 585 nm
D. 694 nm
E. 10,600 nm

**Answers:**

**A.** 308 nm – Incorrect. Excimer lasers deliver ultraviolet B radiation at 308 nm for the depletion of T cells in the epidermis, often in the treatment of autoimmune inflammatory disorders such as psoriasis and vitiligo. T cells are not thought to be involved in the pathogenesis of milia en plaque.
**B.** 532 nm – Incorrect. Potassium titanyl phosphate crystal lasers emit light at 532 nm for absorption by oxyhemoglobin, melanin, and red tattoo pigments in the treatment of cutaneous vascular lesions, dyspigmentation, and tattoos, respectively.
**C.** 585 nm – Incorrect. Light at 585 nm is emitted by pulsed dye lasers and absorbed by hemoglobin for the obliteration of cutaneous vascular lesions. Although having a telangiectatic background, milia en plaque is thought to develop from an eccrine or apocrine, not vascular, etiology.
**D.** 694 nm – Incorrect. Light emitted by ruby lasers at 694 nm targets melanin and some tattoo inks, particularly blues and blacks; traditional use of this wavelength can fade pigmented lesions and depilate skin while Q-switching enables tattoo removal. Melanin is not involved in the pathogenesis of milia en plaque.
**E.** 10,600 nm – Correct. Carbon dioxide lasers can emit light at 10,600 nm, which targets water in the epidermis to induce damage and allow for resurfacing of skin affected by milia en plaque; the associated destruction of adnexal structures may prevent further cyst formation at treated areas.^5^ In addition to surgery, electrodessication, topical retinoids, and tetracyclines may be used in milder cases of milia en plaque, but in instances where plaques are as broad and robust as the one in question, systemic isotretinoin and photodynamic therapy represent other treatment options.

## Conflicts of interest

None disclosed.
